# Automatic imitation of vocal actions is unaffected by group membership

**DOI:** 10.1007/s00426-025-02104-5

**Published:** 2025-03-24

**Authors:** Antony S. Trotter, Hannah Wilt, Patti Adank

**Affiliations:** 1 Department of Neuroimaging, King’s College London, London, UK; 2 Department of Speech, Hearing and Phonetic Sciences, University College London, 2 Wakefield Street, Chandler House, London, WC1N 1PF UK

## Abstract

Converging evidence from behavioural, neuroimaging, and neurostimulation studies demonstrates that action observation engages corresponding action production mechanisms, a phenomenon termed *covert* or *automatic* imitation. Behaviourally, automatic imitation is measured using the stimulus response compatibility (SRC) task, in which participants produce vocal responses whilst perceiving compatible or incompatible speech distractors. Automatic imitation is measured as the difference in response times (RT) between incompatible and compatible trials. It is unclear if and how social group membership, such as the speaker’s sex, affects automatic imitation. Two theoretical accounts make different predictions regarding effects of group membership: the first predicts that automatic imitation can be modulated by group membership, while the second predicts that automatic imitation likely remains unaffected. We tested these predictions for participant sex and distractor sex in an online vocal SRC task. Female and male participants completed an SRC task presenting female or male distractor stimuli. The results show that automatic imitation is not modulated by group membership as signalled by sex. Implications of these results regarding the nature of automatic imitation as a largely stimulus-driven process are considered.

**Supplementary Information:**

The online version contains supplementary material available at 10.1007/s00426-025-02104-5.

## Introduction

Observing someone perform an action engages the neural mechanisms required to perform that action (Buccino et al., 2004; Fadiga et al., 2002), a phenomenon commonly referred to as *covert* or *automatic imitation* (Brass et al., 2000; Cracco et al., 2018). Behaviourally, the stimulus response compatibility (SRC) paradigm is used to investigate links between covert imitation and action production (Heyes, 2005, 2011). SRC tasks have typically been used to study automatic imitation of manual actions (cf. Cracco et al., 2018) but are also used for studying vocal actions. In vocal SRC tasks (Adank et al., 2018; Galantucci et al., 2009; Ghaffarvand Mokari et al., 2020, 2021; Kerzel & Bekkering, 2000; Roon & Gafos, 2015; Wilt et al., 2023; Wilt, Wu, Evans et al. 2024; Wu et al., 2019), participants produce a speech response following a prompt (e.g., say “ba”) while ignoring a distractor (e.g., an audio/video/audiovisual depiction of someone saying “da”). Like manual SRC studies, responses are slower for incompatible (“ba”) than for compatible (“da”) distractors. Automatic imitation effects are defined as the response time (RT) difference between the two compatibility conditions; larger differences indicate more automatic imitation (Heyes, 2011).

As imitation has often been argued to play a role in social behaviours (Chartrand & Bargh, 1999), a key question is whether social factors, such as group membership, modulate the magnitude of automatic imitation. Here, *ingroup* is defined as a social group whose members share one or more personal characteristics (e.g., sex/gender, age, ethnicity, social status, political affiliation, or education level) with an individual, while *outgroup* is a social group whose members do not share such characteristics. Theoretical accounts make contrasting predictions regarding effects of group membership on automatic imitation. Social Top-down Response Modulation (STORM; Wang & Hamilton, 2012) argues that mimicry– capturing a wide range of imitative behaviours - is controlled in a ‘Machiavellian’ fashion to increase social advantage. This theory draws on research suggesting that individuals show greater overt imitation of high social status individuals (Cheng & Chartrand, 2003) and ingroup members (Bourgeois & Hess, 2008; Yabar et al., 2006), and reduced imitation towards outgroup members (Bourgeois & Hess, 2008). STORM predicts that automatic imitation should be increased for members of participants’ ingroup and decreased for participants’ outgroup. In contrast, Associative Sequence Learning (ASL, Catmur et al., 2009; Heyes, 2005), does not make explicit claims with respect to the effect of top-down factors on automatic imitation, and thus remains largely agnostic in this respect. However, ASL posits that automatic imitation may therefore largely be ‘stimulus-driven’, i.e., processed in a bottom-up fashion. ASL posits that visual and motor action components are linked by long-term stimulus response (SR) bonds, whereby perception of an auditory/visual/audiovisual stimulus engages the motor representation (Heyes, 2011). Thus, in an SRC task, an audiovisual link between seeing and hearing a speaker articulate a syllable and the motor representation for executing this syllable are contingent on a direct association between prior action observation and the audiovisual representation.

Several studies examined how group membership affects automatic imitation. Gleibs et al. (2016), participants were assigned to groups that believed they would either work cooperatively or competitively against distractors following a manual SRC task. Imitation effects did not differ between in- and outgroup stimuli in the competitive condition. Larger automatic imitation effects were observed for ingroup targets for participants in the cooperative condition. Genschow et al. (2022) conducted four manual SRC experiments in which group membership was signalled by a coloured glove, or a flag (Germany, China, USA), and two where group membership was signalled by the skin colour of an artificial stimulus. Imitation effects did not differ between in- and outgroup stimuli. De Souter et al. (2021) conducted a manual SRC task to evaluate the effect of in-/outgroup on automatic imitation, by testing the possibility that social group modulations emerge only when people can directly compare in- and outgroup distractors. They ran three experiments in which they measured automatic imitation of two simultaneously shown hands: one in-group and one out-group hand. Group membership was signalled by different coloured gloves (blue or green), attributed to different political affiliations. Participants were assigned an ingroup by telling them that the colours matched with their chosen political affiliation (the colour of the ingroup glove was counterbalanced across participants). However, like Genschow et al., Dr Souter et al. report no effects of group membership on automatic imitation in any of their three experiments.

Other studies used the SRC task to measure effects of distractor sex on automatic imitation. Rauchbauer et al. (2015) presented female and male participants with female distractor stimuli in a manual SRC task also manipulating in-/outgroup (ethnicity) and facial expression (happy or angry) of the distractor. The distractor stimuli consisted of a (happy or angry) female face together with a manual distractor. No effects were found of participant sex on automatic imitation but automatic imitation was increased for the distractor stimuli with a happy facial expression and for out-group distractors (thus opposing STORM’s predictions regarding top-down factors). In Butler et al. (2016), female and male participants completed a manual SRC task with distractor stimuli presenting a female hand, paired with happy, angry, or neutral facial expressions of the distractor. Participants were not explicitly informed that the hand belonged to a woman, but the study showed sex effects: female participants showed larger automatic imitation effects than male participants. This result was argued to either reflect and ingroup bias for the female participants, and an outgroup bias for the male participants, or a sex-based difference related to differences in cognitive mechanisms in male and female participants. Moreover, the result showed increased automatic imitation for the happy facial expression for their Experiment 1. In Genschow et al. (2017), female and male participants conducted a manual SRC task with distractor stimuli presenting a male hand. The results indicated a sex difference, female participants showed more automatic imitation for the outgroup (male) hand. Darda et al. (2020) conducted a more direct test of participant sex on automatic imitation. In Experiment 1, they presented male and female participants with distractor stimuli consisting of a female hand together with of a female face (fearful, angry, happy, sad, or neutral expression) and report increased automatic imitation for female participants only. In Experiment 2, the distractors were changed to independently measure effects of imitative and spatial compatibility, and the speaker’s face was removed, leaving only a female (left) hand. In Experiment 2, spatial compatibility effects were reduced by rotating the hand stimulus 90^o^ clockwise. Here, the results again indicated more automatic imitation for the female participants. In Experiment 3, images of a left and right hand were used to measure imitative and spatial compatibility effects independently. The results showed an increased spatial compatibility effect for female participants, but no sex difference for the imitative compatibility conditions. Finally, Cracco et al. (2018) performed a meta-analysis based on 161 studies and 226 SRC experiments and analysed the results based on a sex split per experiment. They report that automatic imitation is larger when the model’s sex matches the sex of most of the participants, and automatic imitation for accuracy was stronger in predominantly female participant samples than in predominantly male samples. Distractor-participant sex overlap and participant sex thus play a role in moderating automatic imitation. However, a limitation of this meta-analysis is that experiments were classified as having a predominantly female/male sample if > 51% of the participants were female/male, and samples were not entirely single sex based.

Thus, support for STORM is mixed: some studies support STORM’s predictions (Butler et al., 2016; Cracco et al., 2018; Rauchbauer et al., 2015), while others find no effect, thus supporting an agnostic view with respect to top-down influences on automatic imitation from by ASL (Darda et al., 2020; De Souter et al., 2021), and others even report results opposite to STORM’s predictions (Genschow et al., 2017). This lack of support for STORM may be due to limitations of these studies. First, distractor sex might not have been signalled clearly to participants, especially in Darda et al.’s Experiments 2 and 3 and in Butler et al., as distractor stimuli consisted of a hand image. While observers can identify someone’s sex from a hand above chance (Gaetano et al., 2016), faces may be a more reliable cue, as the accuracy of classification of someone’s sex based on a face is close to ceiling (Bruce et al., 1993). Second, participants were not informed about the sex status of the distractor, so it is unclear if they actually perceived the distractor’s sex accurately. Third, no study thus far disentangled effects of distractor sex and participant sex, as all either used a female model or a male model only and sex-specific overlap in automatic imitation could be established for one sex only.

### The current study

We aimed to establish the effect of group membership as signalled by sex on automatic imitation of vocal actions. The use of vocal actions allowed us to more effectively signal the distractor’s sex as in vocal SRC tasks, the stimulus is displayed by the face and/or voice. While some vocal SRC tasks use visual-only stimuli (Kerzel & Bekkering, 2000; Virhia et al., 2019; Wu et al., 2019), here we chose to use audiovisual vocal distractor stimuli (Adank et al., 2018). A person’s sex can also be reliably observed from voice stimuli (Neuhoff, 2017), as the acoustical signatures of male and female voices are different (Titze, 1989). During puberty, the male larynx grows larger and descends further the throat than the female larynx. Acoustically, male puberty results in a lowered fundamental frequency and lowered formant frequencies. Combined face-voice distractor stimuli should therefore provide participants with a stronger, multimodal, signal towards the distractor’s sex compared to hand-only or hand plus (stationary) face stimuli. Moreover, we informed participants of the sex of the distractors at the start of each experimental condition. Finally, we used a 2 × 2 fully crossed factorial between-group design and tested a female and a male participant group with female and male distractor stimuli. This design allowed us to establish if the same-sex bias reported in Cracco et al.’s meta-analysis could be replicated experimentally. STORM predicts that larger imitation effects are expected when participants imitate a member of their ingroup and smaller effects for their outgroup, i.e., someone they perceive to be of the same sex or not, while ASL remains agnostic regarding the effects of group membership on automatic imitation and does not predict a positive effect.

## Methods

### Participants

Recruitment was conducted in two phases: a validation study assessing the quality of participants’ audio recordings, followed by the full SRC task. To obtain our pre-registered minimum sample of 60 participants (30 per group), we initially recruited 221 participants (104 female, 117 male, 18–30 years of age, M = 25.54, SD = 3.30). All declared to be native monolingual speakers of British English residing in the UK at the time of the experiment. All declared to have normal or corrected-to-normal vision, normal hearing and to not have any neurological or psychiatric disorders (including dyslexia). All participants were recruited through Prolific.co. The experiment was hosted on Gorilla.sc (Anwyl-Irvine et al., 2020). Following the validation study, 69 (33 female, 36 male) participants were invited to take part in the main SRC study. Of these, one female and four male participants were excluded due to below chance performance in the SRC task (*n* = 5), and four participants were removed for scoring < 80% accuracy in the catch trials. Note that we omitted to mention this latter requirement regarding this attention check in our pre-registered method. The final sample comprised of 60 participants (30 female, *M*_age_=26.15, *SD*_age_=2.67, 30M, *M*_age_=25.34, *SD*_age_=3.28;). Participants received £5.63 for their time, which is proportionate to £7.50 per hour. The University Research Ethics Committee approved the procedure (UREC #15365.001), and all participants completed an online consent form.

We utilised Bayes’ stopping rule (see Results section for details) to determine our sample size, per our preregistration (aspredicted.org #59734, https://aspredicted.org/xd7ye.pdf). Here, the critical interaction term was the three-way interaction between Participant sex, Stimulus sex and Compatibility. BF_10_ was calculated on the basis of model fit (following Jarosz & Wiley, 2014) for a model including all main effects, two-way interactions and three-way interactions vs. the same model without the critical three-way interaction between Participant sex, Stimulus sex and Compatibility. This procedure was chosen instead of our pre-registered method (comparing a model with all main effects vs. a model with all main effects and the critical three-way interaction) as it was considered more coherent with our backward modelling strategy. If the BF_10_ for the interaction was > 3 or < 0.2, we would regard this as positive evidence in favour of the alternative or null hypotheses respectively. If BF_10_ > 0.2 and < 3.0, we intended to collect an additional four participants and repeat the procedure, which was not needed, as there was strong evidence in favour of the null hypothesis (BF_10_ = 0.018, see Results).

### Materials

**Fig. 1:**
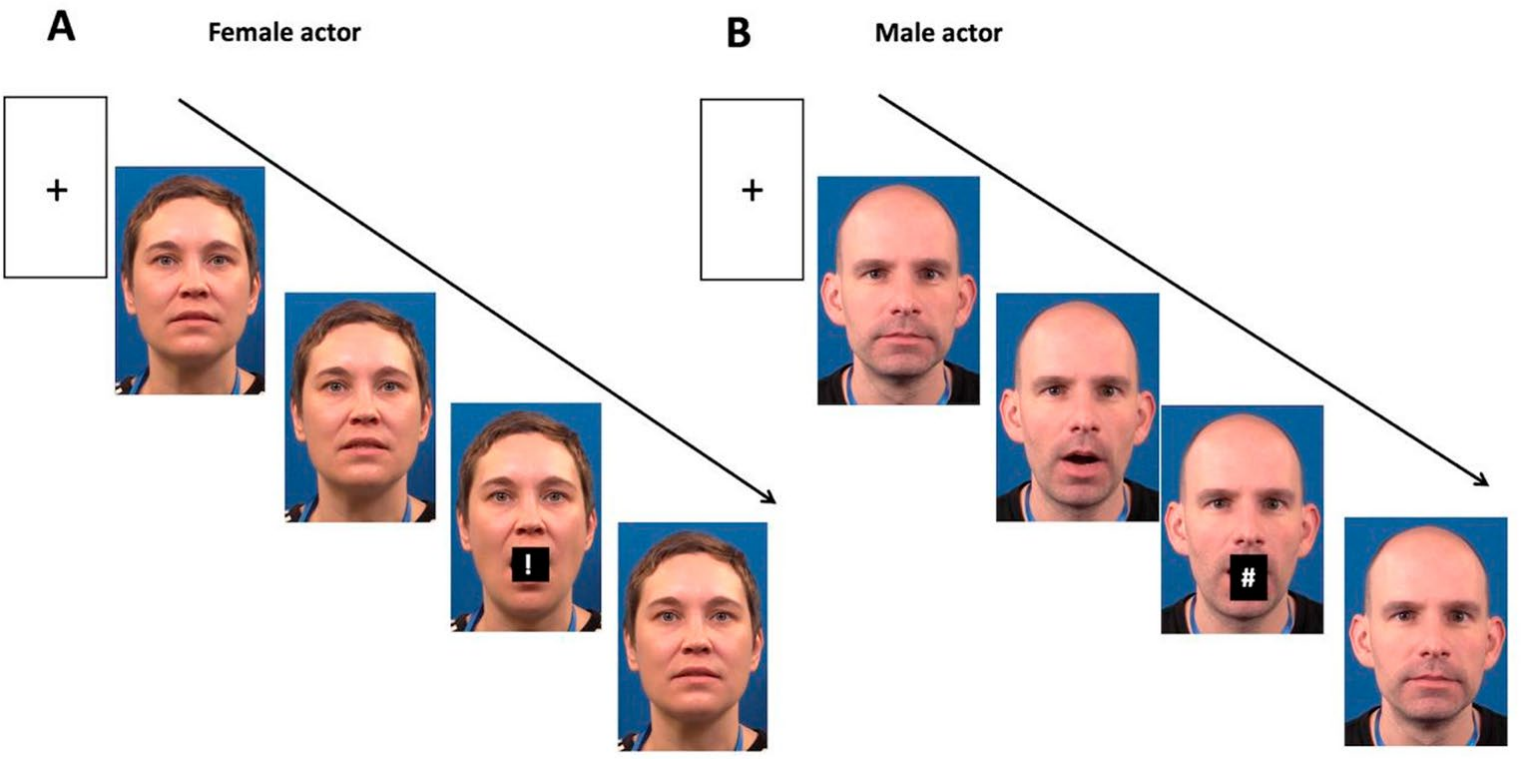
**A** Example of a distractor video in the female condition. The female actor is pronouncing the syllable “da” and the prompt “!” is shown. **B** Example of a distractor video in the male condition. The male actor is pronouncing the syllable “ba” and the prompt “#” is shown

The SRC stimuli were audiovisual clips of a female and a male speaker saying /ba/ and /da/ and the syllable prompts *“#”* and *“!”* (prompt-syllable mappings counterbalanced over participants). Audio and visual speech were recorded simultaneously. The videos (25 fps) were filmed with a Canon Legria HF G30 video camera and edited in Final Cut Pro. Both speakers were shown in colour from their neckline upward (Fig. 1). Imperative prompts were generated in arial font and uniquely positioned for each stimulus to occur between the vermillion borders of the speaker’s lips. In compatible trials, the speaker’s production and prompt matched and in incompatible trials they mismatched. Both videos started and ended with the speaker’s mouth closed in a resting configuration. The onset of articulation, consonant bursts, vowel articulation, cessation of movement, and acoustic characteristics of the distractors can be seen in Table 1. Prompts were presented at one of two SOAs (1,080ms, 1,280ms). Audio stimuli were recorded using a RØDE NO1-A Condenser Microphone and a Focusrite Scarlett 2i4 USB Computer Audio Interface preamplifier plugged into the sound card input of a Dell PC in a sound-attenuated room at 44.1 kHz with 16 bits. Audio recordings were amplitude normalized off-line, down sampled to 22,050 kHz and scaled to 70dB SPL (sound pressure level) using Praat (Boersma & Weenink, 2018) and manually synchronised with the video files. Both audio files had a total duration of 3000ms. The videos were rendered in 1280 × 1080 in.mp4 format.

The catch trial stimuli comprised of audio clips containing two, three or four pure sine tones (250, 275, 325, 350 Hz) lasting 150–200ms, separated by silence (150, 200, 250, 300, 350, 400ms). Each clip started with 1,000ms silence and had a total duration of 3,000ms.

The six headphone check stimuli comprised of a sequence of three sine wave tones. Each 200 Hz tone was played for 1,000ms, with 100ms on- and off-ramps, two at -14dB (in-phase) and one at -20dB (180° out of phase). Each sequence had a duration of 4,000ms (tone duration: 900ms, inter stimulus interval, ISI: 600ms, time before stimulus onset: 100ms, time after last stimulus offset: 100ms).

**Table 1:** SRC stimulus timing information in milliseconds, detailing the onsets of articulation, consonant bursts, vowel articulation, and the cessation of movement.

Sex	Syll	Articulation onset	Consonant burst onset	Vowel onset	Movement offset	Syll dur	Syll f_o_	Syll F_1_	Syll F_2_
F	*Ba*	690ms	1,217ms	1,249ms	1,680ms	578ms	184 Hz	717 Hz	1,231 Hz
F	*Da*	980ms	1174ms	1,215ms	1,680ms	585ms	170 Hz	840 Hz	1,354 Hz
M	*Ba*	1,050ms	1,125ms	1,165ms	1,600ms	504ms	122 Hz	683 Hz	1,067 Hz
M	*Da*	1,060ms	1,093ms	1,139ms	1,600ms	486ms	122 Hz	666 Hz	1,035 Hz

*Note*: Sex indicates whether the speaker was female (F) or male (M). “Syll” indicates the syllable spoken by the speaker, “Dur” indicated duration in millisecond (ms), f_o_ indicates fundamental frequency in Hertz (Hz), F_1_ and F_2_ refer to the first two formants

### Procedure - validation study

The experimental procedure comprised of two main steps. First, a short eligibility screening study was implemented to ensure data quality (cf. Wilt et al., 2023; Wilt, Wu, Evans et al. 2024). Only participants who met the selection criteria for the main task were invited to complete the validation study. An initial 221 participants were invited to the validation study through Prolific.co. Upon receiving an email invitation, participants entered the online study via Gorilla.sc. They were given the study information and provided consent. Participants who did not consent or did not fit the required demographics were rejected. Participants then completed the headphone screening task (Woods et al., 2017). This task was designed to require headphones to complete by manipulating anti-phase attenuation rather than differences in intensity between tones. Participants completed six three-alternative forced-choice (3AFC) trials presenting a sequence of sine tones and were required to indicate which of the trials had the lowest intensity using buttons labelled “FIRST sound is SOFTEST”, “SECOND sound is SOFTEST”, and “THIRD sound is SOFTEST”. Participants had to accurately respond on at least five trials ( > = 83.3%) or they were rejected.

Participants who passed the headphone check subsequently completed the voice test. The voice test was conducted for two reasons; first to ensure that the participants’ self-reported sex matched their vocal responses and to ensure participants’ vocal responses were clearly audible under trial conditions (alongside a simultaneous.mp4 stimulus presentation). However, no participants were excluded based on these two criteria. This task was comprised of four trials which presented a muted SRC stimulus with the speaker’s face obscured by a white bar. 1500ms into the trial, text instructed participants to, “say ba/da”, before a 100ms inter-stimulus interval (ISI). The latency test followed, containing one trial per SRC stimulus (*N* = 8). Participants were instructed to increase their system volume to maximum, place their headphones next to their microphone, and not to respond. Each trial began with a 500ms fixation cross, followed by stimulus presentation and audio recording (3,000ms), then a 100ms ISI. Participants were informed they would be contacted with 48 h to participate in the main study, provided their data was of sufficient quality. See Wilt et al. (2023, 2024a, b) for a description of this latency test and processing of the data.

### Procedure– main task

Upon receiving an email invitation via Prolific, participants entered the online study and were linked to the experiment on Gorilla.sc. They were given the study information and provided consent. Participants who did not consent did not proceed in the experiment. The instructions followed and began with a self-paced SRC trial breakdown. Participants were informed they would see a male or a female speaker. Next, they saw images of the SRC trial with instructions to produce the prompted syllable “*as quickly and accurately as possible”* whilst ignoring the speaker. Next, they were provided two practice trials presenting one female and one male SRC trial. Next, participants were provided with two correctly completed examples of a compatible and an incompatible trial. These trials were created by presenting an audio recording of the experimenter making a “ba” or “da” response 250ms after the imperative prompt. Following these examples, participants completed two additional practice trials. Finally, an explanation of the catch trials was presented, informing participants they would need to count the number of tones in a stimulus, and say the number aloud.

Participants then proceeded to the SRC task. There were six trial blocks (three per level of Stimulus sex). Each block comprised 43 trials and contained only stimulus of a specific speaker (i.e., for each speaker, participants completed 120 trials). Each block started with a catch trial and contained five repetitions of the eight SRC trial types (2 prompts x 2 distractors x 2 SOAs) and two catch trials presented in a randomised order. SRC trials started with a 500ms fixation cross, followed by the SRC stimulus, lasting 3,000ms during which participant vocal responses were recorded, followed by a 100ms ISI. Catch trials started with 500ms fixation cross, followed by a 4,500ms display containing the centrally presented text “*Count the tones then say the number*” during which the stimulus played, and audio was recorded.

After the SRC task, participants completed a latency check, which was identical the one in the validation study, except that each SRC trial type was repeated four times. Participants then took part in a debrief comprising two questions; *“Do you have any feedback on the experiment”* completed with open text response, and *“How would you describe the amount of effort required to complete this experiment?*” which required a scale response from *Very Difficult* (-100) to *Very Easy* (100). On average, participants took ~ 30 min to complete the entire experimental procedure.

### Data recording and analysis

Responses were recorded using Gorilla.sc’s audio recording function using participants’ own hardware at 44.1 kHz with 16 bits, encoded server-side as stereo.weba files, then recoded offline as mono.wav files. Recording was initiated at video onset for 3,000ms. RTs were measured offline. Onsets were determined using the automatic segmentation function of the Prosogram plugin (Mertens, 2014) for Praat (Boersma & Weenink, 2018) checked manually, then extracted using custom scripts and measured relative to prompt onset. To correct for latencies between recording and stimulus onsets, this procedure was followed for the latency check files per Wilt et al. (2023, 2024a, b) with a key contrast: onset of the participant’s production per-recording was measured relative to the onset of articulation in the stimulus. This process resulted in four observations of per-stimulus latency per-participant. Participants’ per-trial RTs were corrected by subtracting the appropriate mean per-stimulus latency. This correction accounted for variance introduced by participant hardware, connection speed, etc. Trials were removed if they were under 2750ms duration (1 trial, 0.01% of total trials). For the SRC task, RTs < 200ms or > 1,300ms were respectively coded as anticipatory or neglected responses (Kerzel & Bekkering, 2000). Note that our pre-registered method stated 1,000ms instead of 1,300ms, we had to adjust the duration due to the overall slower distribution of our RTs collected online compared to the lab-based studies we initially based these RT limits on. Further, trials upon which participants produced the incorrect syllable, multiple, partial, or no responses were coded as errors. Incorrect responses were removed from the RT analysis.

Data analysis utilised separate hierarchical generalised linear mixed effects models (GLMERs) using the *lme4* (Bates & Sarkar, 2005) package for R (R Core Team, 2020), predicting the dependent variables of by-trial RT using a gamma distribution with an inverse link-function (Lo & Andrews, 2015) and by-trial response errors (1 vs. 0) using a binomial distribution and a logit-link function. The present study utilised a mixed between/within design. The analysis proceeded with the within-subjects factors Compatibility (compatible vs. incompatible, -0.5 vs. + 0.5), Stimulus sex (female vs. male; -0.5 vs. + 0.5) and SOA (SOA1 vs. SOA2, -0.5 vs. + 0.5), and the between subject factor of Participant sex (female vs. male; -0.5 vs. + 0.5). Fixed factors were retained in the models if they significantly improved model fit as part of a main effect or interaction. Support for the alternative hypothesis– that group membership modulates automatic imitation– would require substantial evidence for the three-way interaction between Participant sex, Stimulus sex, and Compatibility.

As in Barr et al. (2013), the maximal random effect structure to converge and pass singularity checks was used. Backward selection was conducted to identify the model that best fit the dataset. Starting with higher-order interactions, predictors were removed systematically and chi-squared tests performed using the function *anova()*. Fixed factors were removed from the final model if they did not significantly benefit model fit (*p* >.05) and were not included in any higher-order interactions. At each step, the factor for which there was least evidence of inclusion (i.e., the highest p-value in the chi-squared test) was removed first and the remaining factors reassessed. We stopped when there were no more fixed factors to remove, i.e., when all remaining factors either significantly improved model fit or were included in significant higher-order interactions.

As stated in our pre-registration, in our first analysis (Main analysis) focussed on the key three-way interaction (Participant sex x Stimulus sex x Compatibility) for the RT/error analyses to establish effects of group membership on the automatic imitation of speech. The alternative hypothesis (H_1_) stated that group membership directionally affects automatic imitation. If this is the case, we expected that female participants would show increased automatic imitation for female stimuli and male participants would show increased automatic imitation for male stimuli. This H_1_ required evidence of a three-way interaction between Participant sex, Stimulus sex, and Compatibility. If we could find no evidence for this interaction, we regarded H_0_– that automatic imitation is independent from group membership– as true.

The second analysis (Grouped analysis) collapsed the variables Participant sex and Stimulus sex into a single binary variable indicating whether the stimulus sex matched the participant’s sex: Group (ingroup vs. outgroup, -0.5 vs. 0.5). These analyses therefore predicted by-trial RT/error as a function of the main effects of Group, Compatibility, SOA, and the interactions between these fixed factors. Here, H_1_– that a match between participant sex and stimulus sex affected automatic imitation– required evidence of a two-way interaction between Compatibility and Group.

We utilised sequential hypothesis testing with Bayes factors (Schönbrodt et al., 2017) to determine sample size and assess the likelihood of the null and alternative hypotheses (Jarosz & Wiley, 2014). We will present BF_10_ values to supplement our hierarchical modelling procedure. We obtained BIC values for a model with all main effects, two-way and three-way interactions (alternative) and the same model without the three-way interaction between Participant sex, Stimulus sex and Compatibility (null). We used the difference in BIC to compute BF_10_ using the following equations (Jarosz & Wiley, 2014):

$$\:\varDelta\:BIC={BIC}_{\text{H}1}-{BIC}_{\text{H}0}$$

$$\:{BF}_{01}=\:{e}^{\varDelta\:BIC/2}$$

$$\:{BF}_{10}=1/{BF}_{01}$$

### Transparency and openness

The aims, predictions, design, and proposed analysis of both experiments were preregistered on https://aspredicted.org/xd7ye.pdf. All stimulus materials, R scripts, and raw (text) data can be found on the Open Science Framework https://osf.io/fpbam/.

## Results

### Response times (RTs)

**Fig. 2:**
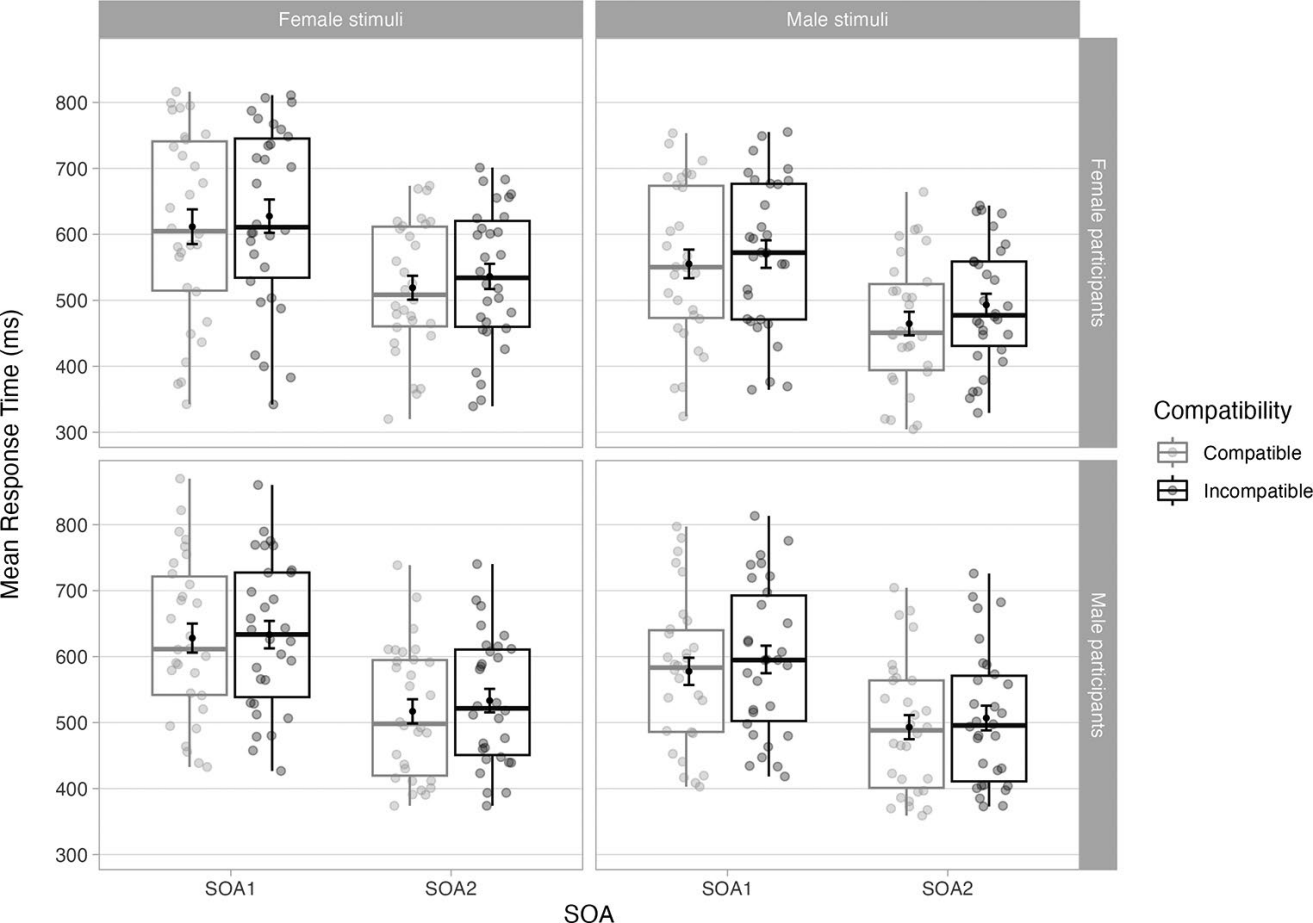
Boxplot illustrating the response times for correct responses by Participant Sex, Stimulus Sex, Compatibility and SOA with mean and +/- 1SE. Note: Points in the background show the raw mean RTs for each participant (points are offset on the x-axis for clarity). The boxplots indicate the first, second (median) and third quartiles, and whiskers indicate 1.5 times the interquartile range of the distribution. Black points in the foreground show the mean and error bars indicate standard errors. SOA = stimulus onset asynchrony

The full dataset for 60 participants contained 14,401 trials. After removing errors (509, or 3.5% of total trials), anticipatory (120, or 0.8% of total trials), and neglected (352, or 2.4% of total) trials, we excluded trials– as per our pre-registration - where RTs exceeded 3 median absolute deviations (MADs) from the participants median RT in each level of load (656, or 4.6% of total trials), leaving 12,855 trials for the analysis. Figure 2 illustrates the descriptive statistics per experimental condition.

### RTs: main analysis

Our first analysis tested the hypothesis that imitation effects are modulated by group membership, as indicated by a significant three-way interaction between Participant sex, Stimulus Sex and Compatibility. Descriptive statistics and the model selection procedure are detailed in Appendices A and B, respectively. The maximal converging random effect structure included by-participant slopes for Stimulus Sex, Compatibility, SOA, as well as for the interactions Stimulus Sex x SOA and Compatibility x SOA. For the fixed effects, all main effects and interactions were included in the final model (Table 2), as the four-way interaction Participant Sex x Stimulus Sex x Compatibility x SOA significantly improved model fit (χ2(1) = 5.91, *p* =.015, BF_10_ = 0.14).

**Table 2:** Final model of Raw reaction times (RTs) in milliseconds (ms) using a gamma distribution and identity link function

Fixed Effect	Estimate	SE	t-value	*p*-value	
**(Intercept)**	**579**	**4**	**155.91**	< 0.001***	
**Participant Sex**	**13**	**4**	**3.22**	0.001**	
**Stimulus Sex**	**-46**	**5**	**-8.81**	< 0.001***	
**Compatibility**	**16**	**2**	**7.36**	< 0.001***	
**SOA**	**-98**	**3**	**-32.98**	< 0.001***	
**Participant Sex x Stimulus Sex**	**22**	**4**	**6.00**	< 0.001***	
Participant Sex x Compatibility	-6	5	-1.11	0.266	
Stimulus Sex x Compatibility	5	3	1.74	0.082	
Participant Sex x SOA	-7	4	-1.97	0.049*	
**Stimulus Sex x SOA**	**17**	**4**	**4.65**	< 0.001***	
Compatibility x SOA	6	3	1.86	0.063	
Participant Sex x Stimulus Sex x Compatibility	-4	3	-1.15	0.249	
**Participant Sex x Stimulus Sex x SOA**	**10**	**6**	**2.13**	0.034*	
Participant Sex x Compatibility Sex x SOA	-3	4	-0.77	0.440	
Stimulus Sex x Compatibility Sex x SOA	2	12	0.14	0.892	
**Participant Sex x Stimulus Sex x Compatibility x SOA**	**-30**	**3**	**-8.87**	< 0.001***	

*Note*: SOA = Stimulus-Onset Asynchrony. * *p* <.05, **p <.01, ***p <.001

The main effect of Compatibility was significant, as RTs were slower in the incompatible condition (*M* = 562ms, *SD* = 200ms) than the compatible condition (*M* = 546ms, *SD* = 123ms), with an average automatic imitation effect of 16ms (*SD* = 23ms). The significant main effect of SOA revealed that RTs decreased from SOA1 (*M* = 600ms, *SD* = 124ms) to SOA2 (*M* = 508ms, *SD* = 100ms).The overarching four-way interaction Participant sex x Stimulus sex x Compatibility x SOA was driven by the fact that at SOA1, compatibility effects in female participants was comparable when perceiving female speaker (*M* = 16ms, *SD* = 32ms) than male speaker (*M* = 15ms, *SD* = 34ms), while male participants showed greater compatibility effects in response to the male speaker (*M* = 18ms, *SD* = 26ms) than to the female speaker (*M* = 5ms, *SD* = 28ms). At SOA2, female participants showed larger compatibility effects in response to the male speaker (*M* = 28ms, *SD* = 26ms) than to the female speaker (*M* = 17ms, *SD* = 18ms), while male participants showed comparable compatibility effects in response to the female (*M* = 16ms, *SD* = 21ms) and to the male speaker (*M* = 14ms, *SD* = 17ms). However, the Bayesian analysis was strongly in favour of the model without the four-way interaction, with BF_10_ = 0.14 indicating that the null hypothesis was 7.34 times more likely than the alternative hypothesis. Hence, this interaction is likely a false positive and should be considered with extreme caution.

To test for the inclusion of the critical three-way interaction Participant sex x Stimulus sex x Compatibility as per our pre-registration (aspredicted.org #59734), a follow-up chi-squared test was conducted comparing a model with all fixed effects, two-way interactions and three-way interactions to the same model but excluding the three-way interaction Participant sex x Stimulus sex x Compatibility. The analysis revealed that the interaction Participant sex x Stimulus sex x Compatibility did not benefit model fit (χ2(1) = 1.82, *p* =.178, BF_10_ = 0.018). The BF_10_ was strongly in favour of the null hypothesis (lower than the pre-registered 0.02 threshold), hence data collection was ended.

### RTs: grouped analysis

**Fig. 3:**
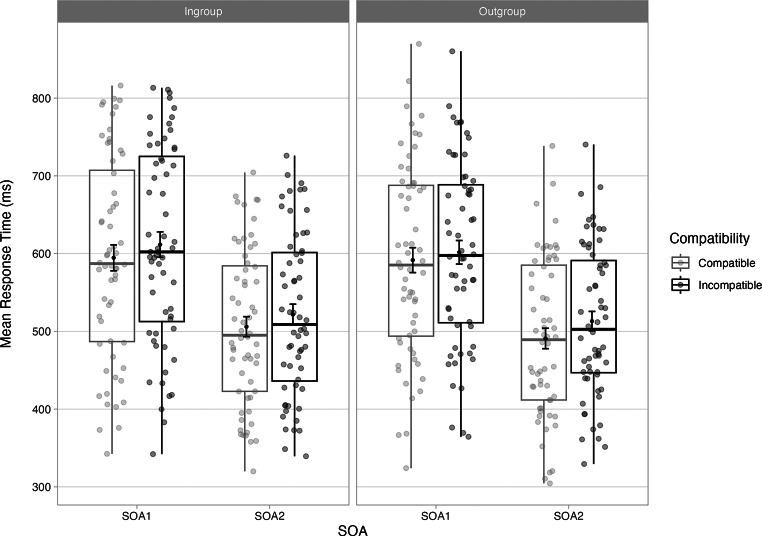
Boxplot illustrating the response times for correct responses by Group, Compatibility and SOA, with mean and +/- 1SE. *Note*: Points in the background show the raw mean RTs for each participant (points are offset on the x-axis for clarity). The boxplots indicate the first, second (median) and third quartiles, and whiskers indicate 1.5 times the interquartile range of the distribution. Black points in the foreground show the mean and error bars indicate standard errors. SOA = stimulus onset asynchrony

The second analysis focused on ingroup vs. outgroup effects by merging the factors Participant sex and Stimulus sex into a single factor Group (ingoup vs. outgroup). Descriptive statistics are displayed in Fig. 3 and in Appendix C, and the backward selection procedure is detailed in Appendix D. The maximal converging random effect model included by-participants slopes for Group, Compatibility and SOA, as well as for the two-way interaction Group x SOA. For the fixed effects, the final model (Table 3) comprised of all main effect Ingroup, Compatibility and SOA, as well as the two-way interaction Group x SOA. Only the main effects were significant. RTs were slower when perceiving a speaker of the same sex (*M* = 558ms, *SD* = 124ms) than a speaker of the opposite sex (*M* = 549ms, *SD* = 120ms). The main effects of Compatibility and SOA are already described in the main RT analysis.

**Table 3:** Final model of Raw reaction times (RTs) in milliseconds (ms) using a gamma distribution and identity link function

Fixed Effect	Estimate	SE	t-value	*p*-value	
**(Intercept)**	**581**	**3**	**215.85**	< 0.001***	
**Group**	**-10**	**2**	**-4.52**	< 0.001***	
**SOA**	**-98**	**2**	**-43.57**	< 0.001***	
**Compatibility**	**17**	**2**	**8.51**	< 0.001***	
Group x SOA	-4	3	-0.15	0.153	

*Note*: SOA = Stimulus-Onset Asynchrony. * *p* <.05, **p <.01, ***p <.001

### Errors: main analysis

14,401 trials were analysed. Descriptive statistics and the backward modelling procedure are detailed in Appendices E and F, respectively. The maximal converging random effect model included by-participants slopes for Participant sex, Stimulus sex, Compatibility and SOA. The final model (Table 4) included main effects Compatibility and SOA, and the two-way interaction Compatibility x SOA.

**Table 4:** Final model of errors using a binomial distribution and logit link function

Fixed Effect	Estimate	SE	t-value	*p*-value	
**(Intercept)**	**-3.11**	**0.12**	**-25.97**	< 0.001***	
**Compatibility**	**0.40**	**0.09**	**4.27**	< 0.001***	
SOA	-0.06	0.12	-0.35	0.624	
**Compatibility x SOA**	**-0.327**	**0.148**	**-2.21**	0.027*	

*Note*: SOA = Stimulus-Onset Asynchrony. * *p* <.05, **p <.01, ***p <.001

The main effect of SOA was not significant. The main effect of Compatibility was significant, with higher error rates (ERs) in incompatible trials (*M* = 7.13%, *SD* = 6.72%) than in compatible trials (*M* = 5.23%, *SD* = 7.30%). This effect was further modulated by SOA, as the difference in ERs between compatible and incompatible trials was larger at SOA1 (*MD* = 2.79%) than at SOA2 (*MD* = 1.00%).

### Errors: grouped analysis

14,401 trials were analysed. Descriptive statistics and the backward modelling procedure are detailed in Appendices G and H, respectively. The maximal converging random effect model included by-participants slopes for Group, Compatibility, and SOA. The final model (Table 5) included main effects of Compatibility and SOA, as well as their interaction. The main effect of Compatibility and its modulation by SOA were significant, these effects are already described in the main error analysis.

**Table 5:** Final model of errors (1 vs. 0) using a binomial distribution and logit link function

Fixed Effect	Estimate	SE	t-value	*p*-value	
**(Intercept)**	**-3.09**	**0.12**	**-26.17**	< 0.001***	
**Compatibility**	**0.40**	**0.09**	**4.24**	< 0.001***	
SOA	-0.05	0.12	-0.46	0.6472	
**Compatibility x SOA**	**-0.33**	**0.15**	**-2.26**	0.024*	

*Note* SOA = Stimulus-Onset Asynchrony. * *p* <.05, **p <.01, ***p <.001

## Discussion

We aimed to establish whether automatic imitation of vocal actions was affected by group membership, operationalised as speaker sex. We conducted an online vocal SRC task with audiovisual (face and voice) female and male distractors and a female and male participant group. The results from the two analyses (Analysis 1: Group Membership, Analysis 2: Sex Match) were as follows. The main analysis showed an overall automatic imitation effect of 16ms, and an effect of SOA where participants responded 98ms faster for SOA2. Moreover, it was found that group membership did not affect automatic imitation, as the critical three-way analysis Participant sex x Stimulus sex x Compatibility was insignificant. The Bayesian analysis further revealed that the H_0_ was 54.60 times more likely to be true than the H_1_ (BF_10_ = 0.018). This analysis revealed a significant four-way interaction Participant sex x Stimulus sex x Compatibility x SOA, it is likely that this effect was a false positive, since the Bayesian analysis strongly favoured the H_0_ as 7.34 more likely to be true than the H_1_ for both interactions (BF_10_ = 0.14). The grouped analysis furthermore demonstrated no support for the crucial two-way interaction between Group and Compatibility.

The main analysis of the errors showed that participants made 1.9% fewer errors for compatible than for incompatible trials. This difference between incompatible and compatible trials was larger at SOA1 (2.79%) than at SOA2 (1.00%). No other effects were found. The results for the grouped analysis were similar, with main effects of Compatibility and an interaction between Compatibility and SOA, but no other effects.

Therefore, the results found no evidence that sex in/outgroup membership modulated automatic imitation, as the main analyses did not reveal a significant three-way interaction between Participant sex, Stimulus sex and Compatibility, and the grouped analyses did not find a significant two-way interaction between Group and Compatibility. In fact, we found decisive evidence for the null hypothesis; namely that group-membership does not affect automatic imitation. Automatic imitation was therefore robust to the effect of group-membership, counter to the predictions of STORM and supportive of the predictions of ASL. Finally, faster RTs (92ms faster) were found for SOA2 compared to SOA1, replicating findings of previous work examining automatic imitation using a vocal SRC task (Adank et al., 2018; Wilt et al., 2023; Wu et al., 2019).

### Is automatic imitation stimulus-driven?

As outlined in the introduction, evidence for effects of top-down manipulations, such as speaker sex, on automatic imitation is mixed. STORM predicts that automatic imitation will be affected by top-down manipulations, while ASL does not make specific predictions and remains largely agnostic. Some (manual) studies find that top-down manipulations affect the size of automatic imitation (Butler et al., 2016; Cracco et al., 2018; Rauchbauer et al., 2015), while others find null effects (Darda et al., 2020), and some oppose STORM’s predictions (Genschow et al., 2017).

In contrast, *stimulus-driven* (or bottom-up) manipulations, such as degradation or simplification of the distractor stimuli, affects automatic imitation effects (e.g., Press et al., 2006; Wilt, Wu, Trotter et al., 2024). Press et al. (2006) presented participants with top-down and stimulus-driven stimulus manipulations in a factorial design, using an opening/closing human or robotic hand. In Experiment 1, robotic stimuli were created by the additional of a wire ‘wrist’ to human hand stimuli. RTs were recorded via electromyography (EMG) from the hand muscles. Press et al. conducted a post-hoc questionnaire, which indicated that the robotic distractor’s movements were less animate than those of the human stimuli. However, the results showed no difference in the size of the automatic imitation effect between human and robotic stimuli. In Experiment 2, the human and robotic stimuli were highly stylised, and the robotic stimuli were more angular and symmetrical than the human stimuli, robotic movements elicited a smaller automatic imitation effect than the human movements. As in Experiment 1, both sets of stimuli were in fact human-generated, but participants believed the robotic stimuli were less human-like. Thus (top-down) beliefs about the animacy of the stimuli did not affect automatic imitation effects.

Wilt et al. (2024a, b) also utilised a manual SRC task to dissociate the effects of top-down and stimulus-driven factors. In Experiment 1, Wilt et al. (2023) presented participants with a human hand and a computer-generated (blue) hand to modulate perceived stimulus animacy as the top-down modulation. The visual clarify of both types of stimuli was also degraded using Gaussian blur to serve as the stimulus driven (bottom-up) factor. Participants were informed that the artificial hands were computer-generated and that some images would be blurry. The results of Experiment 1 showed a smaller automatic imitation effect for visually degraded stimuli, but no effect of the animacy. Experiment 2 modulated stimulus clarity parametrically in five steps and found decreasing compatibility effects for stimuli with lower clarity. Stimulus-driven, but not top-down, factors manipulated automatic imitation. These studies demonstrate that automatic imitation effects are not modulated by participants’ belief regarding the stimulus’ animacy. Overall, these results offer the possibility that stimulus-driven factors may modulate automatic imitation to a larger degree than top-down manipulations.

Finally, it is notable that the only experimental studies to show a positive effect of top-down manipulations on automatic imitation also use emotional facial expressions (Butler et al., 2016; Rauchbauer et al., 2015), where happy facial expressions result in increased automatic imitation. It can be argued that emotional facial expressions do not conclusively represent a top-down manipulation, since as facial expressions may directly influence participants’ perceptions or response without requiring the same level of conscious interpretation that would be required for processing other types of information such as group membership (sex, ethnicity). Facial expression may therefore represent a stimulus-driven manipulation instead of a top-down, or social, manipulation, also because emotional expressions are processed in a subconscious manner to an extent that is not the case for other person-related aspects such as sex (Ohman et al., 2001). Also, as we argue in the next section, top-down manipulations may only increase automatic imitation if participants are placed in an experimental condition in which they expect to cooperate with the distractors (Gleibs et al., 2016).

### Limitations and future directions

First, while our findings are in line with prior literature employing a vocal SRC task (Wilt et al., 2023; Wilt, Wu, Evans et al. 2024), and steps were taken to account for the variability introduced using online testing, it is likely that the response time data contains more extraneous variance than would be found in a lab. Whilst we believe that the combination of our validation study and latency correction procedures paired with the use of the Bayes’ stopping rule should overcome this issue, it would be prudent to replicate the experiment in a lab-based environment.

Second, using online testing also implies we cannot be sure of our participants’ sex status. We asked participants to confirm their sex during the consent and demographics screening stage of the online experiment. No participant was excluded based on their speech recordings. Yet, a disadvantage of online experiments is that it is not feasible to directly interact in the lab with the participants, so therefore, to be sure that participants met all inclusion criteria as closely as possible, the experiment should be replicated in the lab.

Third, our task did not explicitly require the classification of the distractor’s perceived sex in the response, and we also did not implement an explicit manipulation check. Such a check could have been used to verify if participants perceived the female and male distractors as actual in- or out-group members, and/or if they perceived the in-group distractor as more similar and thus may have experienced more affiliation towards them compared to the out-group distractor. The representation of stimulus sex was implied in the distractor’s face and voice, plus the speaker’s sex was announced before each block. It is thus unclear if participants perceived the distractor stimuli as the intended sex. Moreover, asking that the participants to explicitly classify the distractor as female or male might have reinforced the top-down effect of group membership and would have provided a stronger test of the effect of social factors on automatic imitation. Indeed, in studies of the influence of group-membership (e.g., Genschow et al., 2022) on SRC effects, group membership is signalled only by the stimuli, failing to factor into the primary response, under which conditions it fails to affect responses. Genschow et al. conducted four manual SRC experiments in which the group membership of a stimulus was signalled by a coloured glove, or a flag (Germany, China, or USA), and two where group membership was signalled by the skin colour of an artificial stimulus. Contrary to STORM’s predictions, imitation effects did not differ between in- and outgroup stimuli. Further, participants’ feelings of affiliation or perceived similarity to the target failed to modulate the effects. These contrasting findings may suggest that social group membership alone may be insufficient to elicit top-down control of imitation; it may require specific task manipulations to occur, for example, the expectation of cooperation, wherein imitation may improve a social goal. In contrast, Gleibs et al. (2016) utilised a between-groups design wherein participants were assigned to groups that believed they would either work cooperatively or competitively against distractors following the SRC task. Imitation effects did not differ between in- and outgroup stimuli in the competitive condition. Larger imitation effects, critically, were observed for ingroup distractors for participants in the cooperative condition. Notably, in Gleibs et al. the group membership of the stimulus is tied to overall task goals (future cooperation), whilst in Genschow et al. (2022) group membership was secondary. These results suggests that for group membership to modulate imitation, it may need to be tied to the primary task beyond the visual appearance of a stimulus. Overall, therefore, certain preconditions may be necessary to elicit this type of social, top-down control, suggesting that automatic imitation could be conditionally stimulus-driven. Therefore, future experiments could utilise a secondary two alternate forced-choice task wherein after each SRC trial participants are required to identify with a keyboard response whether the stimulus sex matched their own.

Fourth, we cannot be sure that our null effect for speaker sex could be due to the fact we only used two people as models for our distractor stimuli, and that both were relatively similar in terms of hair length (a short crop for the female model and shaven for the male model) and no facial hair such as a beard on the male model. Perhaps the models used counteracted the influence of the predicted group membership effect. Future studies could remedy this issue by adding more models, thereby removing this potential counteract/confound or at least averaging out its potential effects, allowing for a more precise test of the influence of group membership on automatic imitation.

Finally, the null effect in our results for group membership results leave open the possibility that, like automatic imitation of manual actions, automatic imitation of vocal actions may be modulated by bottom-up factors, and not by top-down factors. Yet, this possibility was not tested in the current experiment; the design did not include a stimulus-driven manipulation. A follow-up experiment could incorporate a bottom-up degradation analogous to the clarity degradation in Wilt et al. (2024a, b), e.g., by adding background noise to the speech sound in the video.

## Conclusion

We utilised distractor and participant sex as a pre-existing social category to assess the effects of group-membership on automatic imitation of vocal actions. Our results suggest that, under certain circumstances, automatic imitation is robust to top-down influences, including social factors such as sex, therefore supporting predictions from ASL. This study therefore adds to growing evidence suggesting that automatic imitation of vocal and manual actions should be characterised as a basic sensorimotor associative process that does not necessarily interact with social or cognitive factors.

## Electronic supplementary material

Below is the link to the electronic supplementary material.

Supplementary Material 1

## Data Availability

The aims, predictions, design, and proposed analysis of both experiments were preregistered on https://aspredicted.org/xd7ye.pdf. All stimulus materials, R scripts, and raw (text) data can be found on the Open Science Framework https://osf.io/fpbam/.
