# Supplementary material for: Automatic imitation of vocal actions is unaffected by group membership
Source: Psychol Res. 2025 Mar 24;89(2):74. doi: 10.1007/s00426-025-02104-5 (PMC11933201; doi:10.1007/s00426-025-02104-5)
Supplement: Supplementary file 1 — Supplementary Material 1 [file 426_2025_2104_MOESM1_ESM.docx]

**Appendix A: Descriptive statistics for the main RT analysis**

**Table A1.** Mean reaction times (RTs) and standard deviations (SD) in milliseconds (ms) for each experimental condition.

| Participant sex | Stimulus sex | Compatibility | SOA | RT | SD |
| --- | --- | --- | --- | --- | --- |
| Female | Female | Compatible | SOA1 | 612 | 144 |
|  |  |  | SOA2 | 519 | 99 |
|  |  | Incompatible | SOA1 | 628 | 138 |
|  |  |  | SOA2 | 536 | 105 |
|  | Male | Compatible | SOA1 | 555 | 119 |
|  |  |  | SOA2 | 465 | 98 |
|  |  | Incompatible | SOA1 | 570 | 114 |
|  |  |  | SOA2 | 493 | 93 |
| Male | Female | Compatible | SOA1 | 628 | 121 |
|  |  |  | SOA2 | 517 | 100 |
|  |  | Incompatible | SOA1 | 633 | 114 |
|  |  |  | SOA2 | 533 | 98 |
|  | Male | Compatible | SOA1 | 578 | 113 |
|  |  |  | SOA2 | 493 | 100 |
|  |  | Incompatible | SOA1 | 596 | 114 |
|  |  |  | SOA2 | 507 | 102 |

*Note:* SOA = Stimulus-Onset Asynchrony.

**Appendix B: Backward modelling procedure for the main RT analysis**

The maximal converging random effect structure comprised by-participant slopes for Stimulus sex, Compatibility, SOA, Stimulus sex x SOA, and Compatibility x SOA. Removing the four-way interaction Participant sex x Stimulus sex x Compatibility x SOA led to convergence failures, hence the random effect structure was simplified further. The next converging random effect structure included by-participant slopes for Stimulus Sex. The four-way interaction Participant sex x Stimulus Sex x Compatibility x SOA was found to improve model fit (χ2(1)=5.91, *p*=0.015, BF_10_=0.14) and hence the saturated model was retained. As the saturated fixed effect structure was retained, we chose to report the model with the most complex random effect structure following Barr et al. (2013). A chi-squared test comparing the saturated model with the more complex random effect structure (by-participant slopes for Stimulus sex, Compatibility, SOA, Stimulus sex x SOA, Compatibility x SOA) vs. the simplified random effect structure (by-participant slopes for Stimulus sex) confirmed that the more complex model better fit the data (χ2(18)=518.04, *p*<0.001, BF_10_=3.69 x 10^75^).

**Appendix C: Descriptive statistics for the grouped RT analysis**

**Table C1.** Mean reaction times (RTs) and standard deviations (SD) in milliseconds (ms) for each experimental condition.

| Group | Compatibility | SOA | RT | SD |
| --- | --- | --- | --- | --- |
| Ingroup | Compatible | SOA1 | 595 | 130 |
|  |  | SOA2 | 506 | 100 |
|  | Incompatible | SOA1 | 612 | 127 |
|  |  | SOA2 | 522 | 104 |
| Outgroup | Compatible | SOA1 | 592 | 124 |
|  |  | SOA2 | 491 | 102 |
|  | Incompatible | SOA1 | 602 | 118 |
|  |  | SOA2 | 513 | 96 |

*Note:* SOA = Stimulus-Onset Asynchrony.

**Appendix D: Backward modelling procedure for the grouped RT analysis**

The maximal converging random effect model included by-participants slopes for Group, Compatibility and SOA, as well as for the two-way interaction Group x SOA. The three-way interaction Group x Compatibility x SOA failed to improve model fit and was removed from the model (χ2(1)=0, *p*>.999, BF_10_=0.0006). The two-way interaction Group x Compatibility did not improve model fit and was removed (χ2(1)=1.03, *p*=3.09, BF_10_=0.01), followed by the two-way interaction Compatibility x SOA (χ2(1)=2.78, *p*=0.095, BF_10_=0.03). Removal of the two-way interaction Group x SOA led to convergence failures; hence the interaction was maintained in the model. The final model comprised of all main effect Group, Compatibility and SOA, as well as the two-way interaction Group x SOA.

**Table D1.** Saturated model of reaction times (RTs) gamma distribution and identity function.

| Fixed Effect | Estimate | *SE* | t-value | p-value |
| --- | --- | --- | --- | --- |
| **(Intercept)** | **581** | **17** | **34.42** | **<0.001***** |
| Group | -10 | 26 | -0.40 | 0.691 |
| **Compatibility** | **16** | **9** | **1.90** | **0.058** |
| **SOA** | **-98** | **4** | **-26.58** | **<0.001***** |
| Group x Compatibility | 2 | 4 | 0.39 | 0.697 |
| Group x SOA | -4 | 5 | -0.76 | 0.450 |
| **Compatibility x SOA** | **5** | **2** | **2.23** | **0.026*** |
| Group x Compatibility x SOA | 15 | 11 | 1.41 | 0.159 |

| *Note:* SOA = Stimulus-Onset Asynchrony. * *p*<.05, ***p*<.01, ***p<.001. |
| --- |

**Appendix E: Descriptive statistics for the main error analysis**

**Table E1.** Mean error rates (ERs) and standard deviations (SDs) for each experimental condition.

| Participant sex | Stimulus sex | Compatibility | SOA | ER (%) | SD (%_ |
| --- | --- | --- | --- | --- | --- |
| Female | Female | Compatible | SOA1 | 5.34 | 5.30 |
|  |  |  | SOA2 | 5.11 | 5.59 |
|  |  | Incompatible | SOA1 | 6.56 | 6.69 |
|  |  |  | SOA2 | 6.33 | 6.91 |
|  | Male | Compatible | SOA1 | 4.06 | 5.89 |
|  |  |  | SOA2 | 6.44 | 7.78 |
|  |  | Incompatible | SOA1 | 6.89 | 5.87 |
|  |  |  | SOA2 | 7.22 | 8.49 |
| Male | Female | Compatible | SOA1 | 6.00 | 7.75 |
|  |  |  | SOA2 | 4.44 | 6.69 |
|  |  | Incompatible | SOA1 | 9.22 | 8.87 |
|  |  |  | SOA2 | 6.11 | 6.56 |
|  | Male | Compatible | SOA1 | 4.89 | 7.91 |
|  |  |  | SOA2 | 5.56 | 6.90 |
|  |  | Incompatible | SOA1 | 8.78 | 8.64 |
|  |  |  | SOA2 | 5.89 | 5.72 |

*Note:* SOA = Stimulus-Onset Asynchrony.

**Appendix F: Backward modelling procedure for the main error analysis**

The maximal converging random effect model included by-participants slopes for Participant sex, Stimulus sex, Compatibility and SOA. The saturated model is displayed in Table F1. The four-way interaction failed to improve model fit and was removed from the model (χ2(1)=0.0004, *p*=0.984, BF_10_=0.01). None of the three-way interactions improved model fit, and these were removed in the following order: Participant sex x Stimulus sex x SOA (χ2(1)=0.01, *p*=0.904, BF_10_=0.01); Participant sex x Compatibility x SOA (χ2(1)=0.37, *p*=0.545, BF_10_=0.01); Participant sex x Stimulus sex x Compatibility (χ2(1)=0.48, *p*=0.488, BF_10_=0.01); Stimulus sex x Compatibility x SOA (χ2(1)=2.39, *p*=0.122, BF_10_=0.03). The two-way interaction Participant Sex x Stimulus sex did not benefit model fit (χ2(1)=0.01, *p*=0.924, BF_10_=0.01) and was removed from the model, followed by the two-way interactions Participant sex x Compatibility (χ2(1)=0.49, *p*=0.483, BF_10_=0.01), Stimulus sex x Compatibility (χ2(1)=0.60, *p*=0.438, BF_10_=0.01), Stimulus sex x SOA (χ2(1)=1.94, *p*=0.164, BF_10_=0.02), Participant sex x SOA (χ2(1)=2.33, *p*=0.127, BF_10_=0.02). The two-way interaction Compatibility x SOA improved model fit (χ2(1)=4.67, *p*=0.031, BF_10_=0.01) and was maintained in the model. The main effect Participant sex failed to improve model fit and was removed (χ2(1)=0.06, *p*=0.800, BF_10_=0.01), followed by the main effect Stimulus sex (χ2(1)=0.06, *p*=0.801, BF_10_=0.0004).

**Table F1.** Saturated model of Errors (1 vs. 0) using a binomial distribution and logit link function.

| Fixed Effect | Estimate | *SE* | t-value | p-value |
| --- | --- | --- | --- | --- |
| **(Intercept)** | **-3.11** | **0.12** | **-26.00** | **<0.001***** |
| Participant sex | 0.04 | 0.23 | 0.18 | 0.857 |
| Stimulus sex | 0.02 | 0.12 | 0.15 | 0.883 |
| **Compatibility** | **0.41** | **0.10** | **4.25** | **<0.001***** |
| SOA | -0.06 | 0.11 | -0.49 | 0.626 |
| Participant sex x Stimulus sex | 0.03 | 0.23 | 0.14 | 0.886 |
| Participant sex x Compatibility | 0.11 | 0.16 | 0.65 | 0.514 |
| Stimulus sex x Compatibility | 0.11 | 0.15 | 0.74 | 0.458 |
| Participant sex x SOA | -0.29 | 0.21 | -1.39 | 0.163 |
| Stimulus sex x SOA | 0.26 | 0.15 | 1.68 | 0.093 |
| **Compatibility x SOA** | **-0.33** | **0.15** | **-2.24** | **0.025*** |
| Participant sex x Stimulus sex x Compatibility | -0.19 | 0.29 | -0.68 | 0.499 |
| Participant sex x Stimulus sex x SOA | -0.03 | 0.29 | -0.12 | 0.905 |
| Participant sex x Compatibility sex x SOA | -0.18 | 0.29 | -0.62 | 0.537 |
| Stimulus sex x Compatibility sex x SOA | -0.46 | 0.28 | -1.63 | 0.104 |
| Participant sex x Stimulus sex x Compatibility x SOA | -0.01 | 0.57 | -0.02 | 0.983 |

| *Note:* SOA = Stimulus-Onset Asynchrony. * *p*<.05, ***p*<.01, ***p<.001. |
| --- |

**Appendix G: Descriptive statistics for the grouped error analysis**

**Table G1.** Mean error rates (ERs) and standard deviations (SDs) for each experimental condition.

| Group | Compatibility | SOA | ER (%) | SD (%) |
| --- | --- | --- | --- | --- |
| Ingroup | Compatible | SOA1 | 5.11 | 6.68 |
|  |  | SOA2 | 5.33 | 6.17 |
|  | Incompatible | SOA1 | 7.67 | 7.75 |
|  |  | SOA2 | 6.11 | 6.29 |
| Outgroup | Compatible | SOA1 | 5.03 | 6.89 |
|  |  | SOA2 | 5.44 | 7.26 |
|  | Incompatible | SOA1 | 8.06 | 7.55 |
|  |  | SOA2 | 6.67 | 7.54 |

*Note:* SOA = Stimulus-Onset Asynchrony.

**Appendix H: Backward modelling procedure for the grouped error analysis**

The maximal converging random effect model included by-participants slopes for Group, Compatibility and SOA. The saturated model is displayed in Table H1. The three-way interaction Group x Compatibility x SOA failed to improve model fit (χ2(1)=0.0005, *p*=0.982, BF_10_=0.01) and was removed from the model. The two-way interaction Group x SOA did not benefit model fit (χ2(1)=0.01, *p*=0.926, BF_10_=0.01) and was removed from the model, followed by the two-way interaction Group x Compatibility (χ2(1)=0.29, *p*=0.589, BF_10_=0.0001). The two-way interaction Compatibility x SOA improved model fit (χ2(1)=4.85, *p*=0.028, BF_10_=0.10) and was kept in the model. The main effect Group did not improve model fit (χ2(1)=0.33, *p*=0.563, BF_10_=0.01) and was removed.

**Table H1.** Saturated model of Errors (1 vs. 0) using a binomial distribution and logit link function.

| Fixed Effect | Estimate | *SE* | t-value | p-value |
| --- | --- | --- | --- | --- |
| **(Intercept)** | **-3.11** | **0.12** | **-25.96** | **<0.001***** |
| Group | -0.08 | 0.12 | -0.64 | 0.521 |
| **Compatibility** | **0.40** | **0.09** | **4.23** | **<0.001***** |
| SOA | -0.05 | 0.12 | -0.45 | 0.651 |
| Group x Compatibility | 0.08 | 0.15 | 0.56 | 0.574 |
| Group x SOA | 0.01 | 0.15 | 0.09 | 0.926 |
| **Compatibility x SOA** | **-0.33** | **0.15** | **-2.26** | **0.024*** |
| Group x Compatibility x SOA | 0.01 | 0.28 | 0.02 | 0.982 |

| *Note:* SOA = Stimulus-Onset Asynchrony. * *p*<.05, ***p*<.01, ***p<.001. |
| --- |
